# Effects of Teucrium Polium Aerial Parts extract on oral glucose tolerance tests and pancreas histopathology in Streptozocin-induced diabetic rats

**Published:** 2012

**Authors:** Mohsen Tatar, Durdi Qujeq, Farideh Feizi, Hadi Parsian, Alieh Sohan Faraji, Sohrab Halalkhor, Roya Abassi, Zeinab Abedian, Roughayeh Pourbagher, Seyed Mohsen Aghajanpour Mir, Hamed Mir, Nayer Seyfizadeh

**Affiliations:** 1 *Cellular and Molecular Biology Research Center (CMBRC), Babol University of Medical Sciences, Babol, Iran*; 2 *Department of Biochemistry & Biophysics, Faculty of Medicine, Babol University of Medical Sciences, Babol, Iran*; 3 *Department of Anatomical Sciences, Faculty of Medicine, Babol University of Medical Sciences, Babol, Iran*

**Keywords:** Teucrium polium, oral glucose tolerance tests, pancreas histology, streptozocin, diabetic rats

## Abstract

Teucrium polium can reduce serum glucose. There are few reports in the literature related to this subject and the resolution of this mechanism requires further experiments. The aim of the present study was to evaluate the effects of Teucrium polium aerial parts extracts on oral glucose tolerance tests and pancreas histology in streptozocin-induced diabetic rats. In order to prepare the aqueous concentrate, aerial parts extract was dissolved in distilled water and was boiled for 30 minutes. For the preparation of ethanolic solution, powder was dissolved in ethanol and mixed by a shaker. Diabetic rats were induced with single IP injection of streptozotocin (STZ) at a dose of 50 mg/kg body weight dissolved in normal saline just before use to the 16 hr fast rats. Both groups, diabetic and normal were sacrificed by ether anesthesia. The tissue samples were formalin fixed and paraffin embedded for microscopic examination in accordance with routine laboratory procedures. Blood was collected from the tail vein of the rats. Serum glucose levels were then measured by commercial kits by using a glucose oxidized method. There were no biochemical abnormalities or histological changes in the pancreas of control rats. Post treatment of Teucrium polium aerial parts extract reduced the severity of streptozotocin diabetic pancreases. Our histopathological investigation along with the biochemical evaluations showed a significant effect on histological changes in the pancreas of induced diabetic rats upon Teucrium polium aerial parts extract treatment (P<0.05).

It was previously shown that Teucrium polium crude extract chemical-induced diabetic rats can significantly decrease serum glucose values (1,2). It has also been demonstrated that administration of a hydro-alcoholic extract of Teucrium polium by gavages enhances serum insulin level in rats (3). As reported by investigators extract of Teucrium polium has hypoglycemic activity in diabetic animals (4). Previous work has indicated that the combinations of Teucrium polium with anticancer drugs may be able to increase capability of cancer chemotherapies. There is accumulating evidence that streptozocin-induced diabetes resulted in a decrease in the number of pancreatic islets (5,6). 

In a recent investigation the possibility of the islets regeneration upon plant extract treatment was studied. However, any significant improvement in lipid metabolism was not shown after Teucrium polium extract treatment (7). In literature , it was reported that administration of medical plants might be useful in preventing hyperglycemia in rabbits (8). Some data have revealed that traditional medicine can be hepatotoxic (9). Many investigators have shown that Teucrium polium extract reduces blood glucose via mechanisms such as enhancement of peripheral metabolism of glucose rather than an increase in insulin release (10). Previous study reported that the aqueous extract of Teucrium polium reduced the serum levels of lipids in rats (11). Evidence revealed that methoxylated flavonoids exhibited a lesser antioxidant activity (12). 

Based on the mentioned studies, it may therefore be interesting to evaluate the effects of Teucrium polium aerial parts extracts on oral glucose tolerance tests and pancreas histology in streptozocin-induced diabetic rats. The present experiments were undertaken to evaluate the effects of Teucrium polium aerial parts extracts on biochemical and histological parameters.

## Materials and Methods

**Plant Materials**

In order to prepare the aqueous form, 100 g of dry Teucrium polium aerial parts were dissolved in two litres of distilled water and were boiled (Behdad –water bath) for 30 minutes. For the preparation of ethanolic solution, 100 g of dry powder was dissolved in one litre of 96 degree 65% ethanol and mixed by a shaker (Labtron –LS -100) for 24 h at room temperature. Both aqueous and ethanol solutions were kept under hood four days to let the solvents evaporate. Finally, the extract was stored at -20 ºC.

**Diabetic induction**

The rats were not fed 16 h before injection. The streptozotocin (STZ) solution was prepared and 0.6ml volume with 50 mg/kg dose was injected intraperitoneally to the rats in the hyperglycemic group. The rats with fasting glycemia more than 250 mg/dl were used as diabetic group. In addition, 0.7 ml normal saline was injected to rats in the control group.

**Animal experiments**

A group of 6 rats was considered as the normal group that received normal chow and tap water. The other 18 hyperglycemic animals were divided randomly and equally into three groups of six. The first and second group of diabetic rats (diabetic experimental rats) received the aqueous Teucrium polium aerial parts extract twice a day through a gavage tube for a period of 14 days. The third group of diabetic rats (diabetic control rats) received only water and rat chow ad libitum. 

**Glucose tolerance test**

At the end of the gavage feeding of the extract, half of the rats in each of the two groups were fasted for 14 hours and underwent oral glucose tolerance test (GTT), while the other half were immediately sacrificed by decapitation. Glucose tolerance test in fasted animals was performed on 16 h fasted rats using one gram glucose/kg body weight. Duplicate blood samples from the animal tails were collected. In all groups, blood was collected by tail snipping at 0, 30, 90 and 120 minutes after glucose load. The glucose concentration data were used to compare glucose tolerance in the various groups.

**Histopathologic evaluation**

The animals in four groups were sacrificed by ether anesthesia. Whole pancreas was dissected. Tissue samples from the pancreas were fixed in formalin %10 and paraffin embedded for microscopic examination in accordance with routine laboratory procedures. A pathologist who was not aware of the experimental treatment counted the islets in each section expressed per cm2**.** Histopathologic examination and grading were carried out on hematoxiline and eosine, stained sections at 5-μm thickness were used for morphometric analyses. 

**Statistical analysis.**

All tests were carried out in triplicate. Statistical analyses were done using SPSS version 16.0. The significance of differences between the mean values were determined by analysis of variance (ANOVA), and a P value of less than 0.05 was considered statistically significant.

## Results

**Fig 1:**
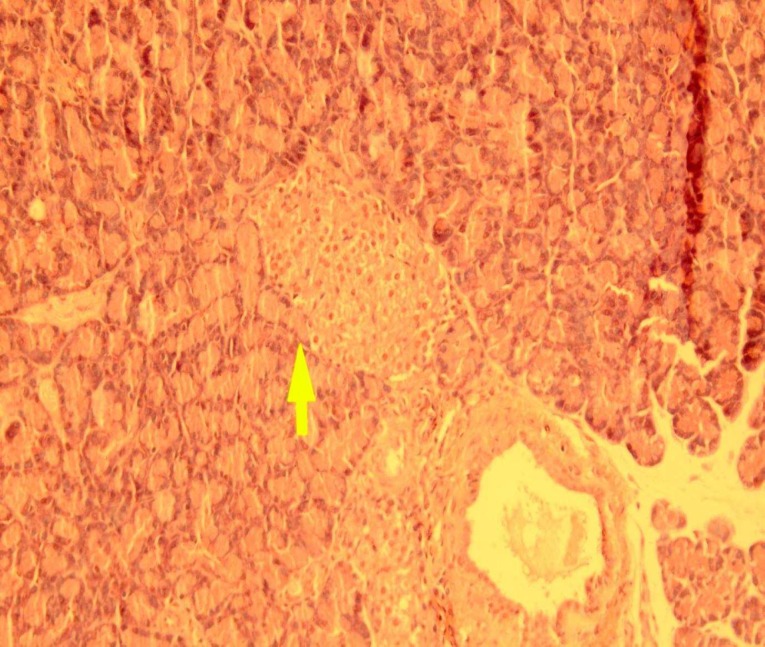
Representative picture of pancreas histology from control rat (hematoxin and eosin, original magnification ×40)

In the present study, it is clearly demonstrated that the pancreas from control rat shows normal pancreatic histoarchitecture islet. There were no abnormalities in the pancreas of control rats. Representative pictures of pancreas histology is shown in Fig.1. 

The pancreas from the diabetic rats showed degeneration of serous acini and the numbers of islet and islet cells were found to be lower than the control group. 

**Fig 2:**
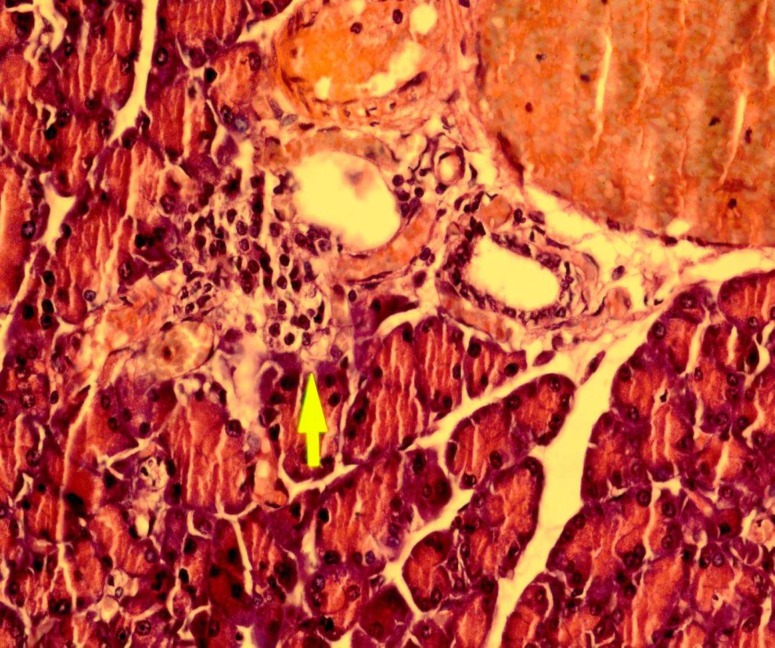
Representative picture of pancreas histology from a diabetic rat ( hematoxin and eosin, original magnification ×40)

As it is evident from fig 2, the pancreases are irregularly shaped and relatively small. Representative picture of pancreas histology is shown in Fig.2.

**Fig 3:**
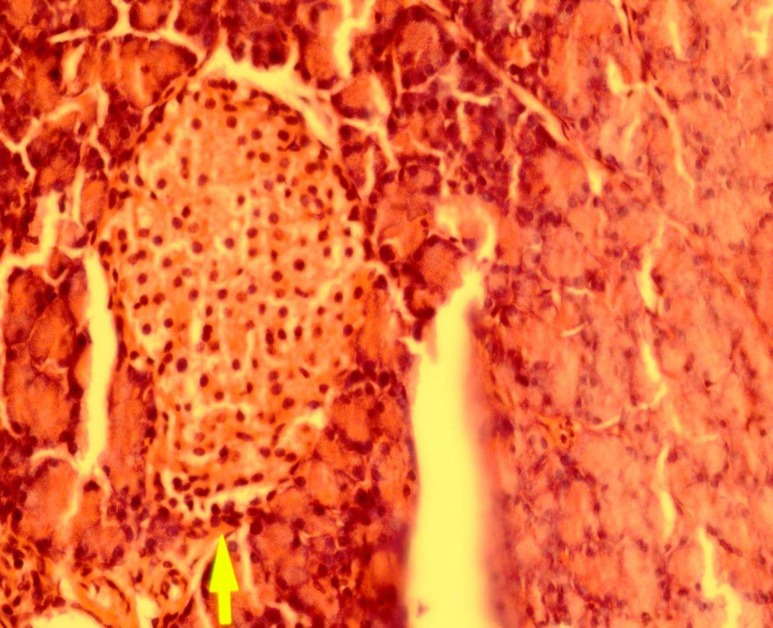
Representative picture of pancreas histology from a diabetic rat treated with ethanolic Teucrium polium aerial parts extract (hematoxin and eosin, original magnification ×40).

The pancreas from a diabetic rat treated with powder of ethanolic Teucrium polium aerial parts extract shows slight to moderate regeneration. Picture of pancreas histology is shown in Fig 3.

**Fig 4:**
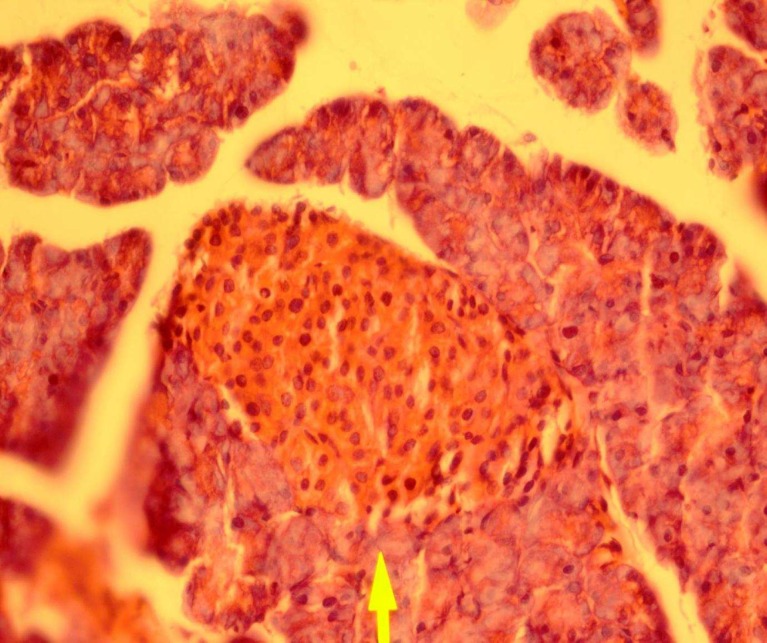
Rrepresentative picture of pancreas histology from a diabetic rat treated with aqueous Teucrium polium aerial parts extract (hematoxin and eosin, original magnification ×40).

The pancreas from a diabetic rat treated with powder of aqueous Teucrium polium aerial parts extract shows moderately regeneration and the inflammatory infiltration has disappeared. Picture of pancreas histology is shown in Fig.4. 

**Fig 5:**
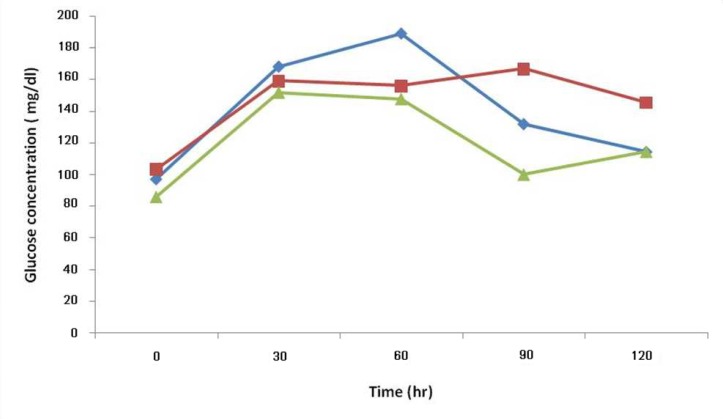
The effects of the aqueous extract of the aerial parts of T. polium on oral glucose tolerance tests in streptozocin-induced diabetic rats.All values have been presented as mean ± standard error, P<0.05. (;● control group, ; ▪ first diabetic group, ♦; second diabetic group).

Animals treated with Teucrium polium aerial parts extract showed normal histology of pancreatic tissue. The effects of the aqueous extract of the aerial parts of Teucrium polium on oral glucose tolerance tests streptozocin-induced diabetic rats is shown in Fig.5. 

These observations indicate that powder of Teucrium polium aerial parts extract prevents the cellular damage of pancreas .

## Discussion

One of the major findings of this study is that the histopathological investigation along with the biochemical evaluations demonstrated the possibility of the pancreatic tissue regeneration upon Teucrium polium aerial parts extract treatment. The regeneration of the pancreas of the streptozotocin -destructed islets is probably due to the fact that pancreas contains stable cells which have the capacity of regeneration. In this regard, Teucrium polium aerial parts extract adminstration may be mobilizes progenitors cell into injured pancreatic tissue. On the other hand, progenitor cells may be participate in this repair mechanism. However, the source and nature of these progenitor cells was not determined in this study. 

Our present findings, taken together with previous results may support the hypothesis that pancreas regeneration is made by the Teucrium polium aerial parts extract. 

The Fig 2, shows a typical pancreatic section from a streptozocin-induced diabetic animal and figures 3,4 show a pancreatic section from a diabetic rat treated with the Teucrium polium aerial parts extract. The number of pancreatic islets per unit area was higher in extract-treated diabetic rats, 65% more than control group. The number of pancreatic islets per unit area significantly decreased in diabetic animals and extract treatment resulted in the regeneration of these islets to the normal range. Our results are in accordance with what showed that Teucrium polium aerial parts extract decreased the serum glucose level of diabetic rats (3,6,7). 

Some limitation and methodological flaws of our study that should be mentioned. Our small sample size might have led to loss of the power of statistical analysis. Our result represents effects of Teucrium polium aerial parts extract on oral glucose tolerance test and regeneration of pancreatic tissue may be some difference from the other investigators findings. We suggest that Teucrium polium aerial parts extract effects on pancreatic function of strptozotocin diabetic rats , but other investigators reported the hepatotoxicity associated with hypoglycemic effects of Teucrium polium .

We conclude that Teucrium polium aerial parts extract stimulate pancreas repair and may be clinically beneficial as an agent to restore or maintain pancreas tissue after injury. The results of this paper can encourage clinical studies to evaluate the potential benefit of Teucrium polium aerial parts extract administration. 
